# Transactional sex amongst young people in rural northern Tanzania: an ethnography of young women's motivations and negotiation

**DOI:** 10.1186/1742-4755-7-2

**Published:** 2010-04-29

**Authors:** Joyce Wamoyi, Daniel Wight, Mary Plummer, Gerry Hilary Mshana, David Ross

**Affiliations:** 1 National Institute for Medical Research, Mwanza, Tanzania; 2 African Medical Research Foundation, Mwanza, Tanzania; 3 Medical Research Council, Social and Public Health Sciences Unit, 4 Lilybank Gardens, Glasgow G12 8RZ, UK; 4 London School of Hygiene and Tropical Medicine, Keppel Street, London WC1E 7HT, UK

## Abstract

**Background:**

Material exchange for sex (transactional sex) may be important to sexual relationships and health in certain cultures, yet the motivations for transactional sex, its scale and consequences are still little understood. The aim of this paper is to examine young women's motivations to exchange sex for gifts or money, the way in which they negotiate transactional sex throughout their relationships, and the implications of these negotiations for the HIV epidemic.

**Method:**

An ethnographic research design was used, with information collected primarily using participant observation and in-depth interviews in a rural community in North Western Tanzania. The qualitative approach was complemented by an innovative assisted self-completion questionnaire.

**Findings:**

Transactional sex underlay most non-marital relationships and was not, *per se*, perceived as immoral. However, women's motivations varied, for instance: escaping intense poverty, seeking beauty products or accumulating business capital. There was also strong pressure from peers to engage in transactional sex, in particular to consume like others and avoid ridicule for inadequate remuneration.

Macro-level factors shaping transactional sex (e.g. economic, kinship and normative factors) overwhelmingly benefited men, but at a micro-level there were different dimensions of power, stemming from individual attributes and immediate circumstances, some of which benefited women. Young women actively used their sexuality as an economic resource, often entering into relationships primarily for economic gain.

**Conclusion:**

Transactional sex is likely to increase the risk of HIV by providing a dynamic for partner change, making more affluent, higher risk men more desirable, and creating further barriers to condom use. Behavioural interventions should directly address how embedded transactional sex is in sexual culture.

## Introduction

The exchange of sex for money or gifts in sub-Saharan Africa has been widely reported. It is generally interpreted as a consequence of women's poverty and economic dependence on men (e.g [1-6]). Many have noted that impoverishment deters women from negotiating safer sex [7-10] and makes younger women vulnerable to the enticements of older men or 'sugar-daddies' [3,9-12].

However, several detailed studies have suggested that material exchange for sex (or 'transactional sex') is not always engaged in through immediate material need. Many Senegalese prostitutes in the Gambia were reported to be from non-impoverished families [13], while Tanzanian Haya women practising prostitution were reported to be both poor and relatively well-off [14]. In southern Uganda, secondary school girls were reported to exchange sex to pay for necessities their parents cannot afford, but half those in a qualitative study said that, whatever their affluence, they would not have sex for free. This would be humiliating since the gift 'rubs off the cheapness of being used' [15]. In Mwanza, Tanzania, girls are said to negotiate sexual deals to their own advantage [16], and in Dar es Salaam, many young women who had experienced abortions were found to be 'active social agents, entrepreneurs who deliberately exploit their partner(s)' [17], and no self-respecting woman would have sex for free. Hunter argues that transactional sex in KwaZulu-Natal is attributable to gendered material inequalities, a particular construction of masculinity, but also 'the agency of women themselves' [3], while Leclerc-Madlala [18] argues that in Durban women see transactional sex as a 'normal' part of sexual relationships motivated to acquire the commodities of modernity. A recent study contrasted policy makers' views of transactional sex in rural Malawi, as driven by survival needs, with the views of the rural women themselves, who said that they are also motivated by attractive consumer goods, passion and revenge [19]. In a review of both quantitative and qualitative studies of age and economic asymmetries in young women's sexual relationships, Luke concluded that:

'girls have considerable negotiating power over certain aspects of sexual relationships with older men, including partnership formation and continuation; however, they have little control over sexual practices within partnerships, including condom use and violence.' [10] (pg 67).

As de Zalduondo and Bernard have argued, attributing transactional sex to economic adversity:

'implies an apology for sexual-economic exchange where none is needed. ....The inference that all instances of sexual-economic exchange are inherently demeaning (and thus probably involuntary) seems to underlie an undifferentiated treatment of the topic in the public health literature.' [20] (pg158)

An anthropological review noted the 'predominantly neutral' attitudes to prostitution in sub-Saharan Africa, and 'a relatively instrumental view of sex within marriage... It is the filiation of children rather than payment in cash which distinguishes wives, prostitutes and others.' [21] (pg 424). This fits Caldwell et al.'s overview [22] that 'sex is seen as a service which women render to men in return for cash and support.... ' (summarised by Heald [23]: 490).

The tendency for the issue of sexual exchange to become polarized, in particular given the 'essentialisations' spawned by debate over Caldwell's 'African sexuality' thesis [24], makes it easy to overlook that there are generally several, overlapping motivations for sex. Although this paper is concerned with material motives, we do not want to suggest that, if they exist, they are to the exclusion of other motives such as physical pleasure, reproduction, self-esteem, love or establishing and maintaining relationships for other non-material reasons. Setel's [25] ethnography from Kilimanjaro Region, Tanzania, provides a detailed analysis of how these diverse motives shape sexual relationships.

Until the last decade, most research in sub-Saharan Africa on sexual transaction focussed on urban areas and commercial sex work [21], rather than transactional sex in rural areas, yet the majority of the population are rural. In Tanzania, for example, 70% of people live in rural areas [26]. Furthermore, most qualitative research on young people's sexual relationships has been conducted with secondary school students (e.g. [1,15,27,28], yet in East Africa only 5% to 25% of young people reach secondary school [29] and they are likely to be highly untypical in terms of relative affluence and ways of thinking about the future. In Tanzania, as a whole, the figure is 6% for males and 5% for females [29], and even lower in rural areas. Most qualitative studies collected data through group discussions, which may bias findings towards normative beliefs [9,10], and we are only aware of two other studies employing any participant observation [18,19].

With quantitative studies the validity of sexual behaviour data is highly problematic [10,30,31], and must be treated very cautiously. Furthermore, survey questions rarely investigate the type of gifts provided or the context of gift giving [10]. This means we have little idea about the proportion of relationships that involve transactions, and to what extent the transactions are specific inducements for sexual access [10].

The aim of this paper is to examine young rural women's motivations to exchange sex for gifts or money, the way in which they negotiate transactional sex throughout their relationships, and the implications of these negotiations for the HIV epidemic. The findings come from an ethnography of young people's sexual behaviour in rural Mwanza Region, northern Tanzania. Most of the data come from participant observation with young people who had not attended secondary school, and most were unmarried.

### Conceptual framework

Although we recognise that sexual relationships are complex phenomena influenced by a multitude of factors at macro-social, micro-social, psychological and physiological levels [32], this paper is restricted almost entirely to social factors. At a macro-level the social factors shaping sexual relationships generally give men greater power than women and create the material and ideological conditions that encourage transactional sex. Although interrelated, they can be broadly divided between economic factors, kinship factors and normative factors. At a micro-level the factors shaping the relative bargaining power of (potential) sexual partners can be divided between their individual attributes, which generally persist over time, and the specific circumstances of a particular sexual encounter. At this level the different dimensions of power sometimes benefited women. The different levels of influence are summarised below, more space being given to the macro-social factors since the main micro-social factors are presented in the Findings section.

Economic conditions in sub-Saharan Africa generally give men far greater power than women. Women rarely hold land in their own right [33], they generally work much longer hours than men [34] but largely because of their domestic responsibilities are far less able to sell their labour. Consequently most are economically dependent on men [7]. The gendered division of labour extends to most areas of work, except for certain farming activities [25,35], and whilst it gives women power in specific spheres [36], in general it greatly benefits men, which some men recognise [7].

However, these patriarchal relations have been being eroded for over a century. While the influence of missionaries on marriage patterns is contested [37], there is no doubt that the increasing dominance of the cash economy has undermined the land or cattle-based power of male elders [24,36,38]. Men's employment initially reinforced their economic power, but in recent decades contraction of formal employment has left men unable to fulfil their newly acquired 'bread-winner' role, undermining their status as head of household [38,39]. Meanwhile women's entrepreneurial skills and harder work give them an advantage in the informal sector, reducing their economic dependence on men and the rationale for marriage [24,36,38-40]. These social changes are almost certainly at their most advanced in urban areas and may only be starting in rural areas dominated by subsistence farming.

Systems of kinship and marriage have been important underlying factors in women's disempowerment [33]. The Sukuma are very similar to their southern neighbours the Nyamwezi, for whom 'rights in the productive and reproductive capacity of women are in large part controlled by and transferred for payment between men.' [41] (pg 72). Bridewealth still determines the nature of marriage, most importantly giving the groom rights to the children, but it also, as in Botswana, 'encompasses the idea that a man has 'paid' for sexual access to the wife' [42] (pg379). It involves a significant transfer of wealth, particularly if the bride is young and considered virtuous, typically six cattle or, increasingly, the cash equivalent (cf. [43]. Since it is paid by the groom's father to the brides' father, it gives them considerable influence over their children's' unions [44]. However, with the socio-economic changes eroding patriarchal control, alternatives to formal marriage, such as *kutoroshwa *(elopement), have become increasingly prevalent [25,45], reducing children's economic dependence on parents [36,46]. Furthermore, in the towns and increasingly in rural areas some women feel able to make strategic choices not to get married at all [36,38-40].

The most relevant social norms relate to women's status and sexual culture. In general, women are of lower social status and are culturally inhibited from asserting their interests in public [7,33]. The predominant sexual culture for young people in rural Mwanza has previously been described in terms of contradictory norms [46]. These ideal standards of behaviour are not entirely prescriptive but can be seen as resources that can be drawn on to legitimate behaviour. Sexual activity is constrained by norms of school pupil abstinence, female sexual respectability and taboos around the discussion of sex. However, these norms are incompatible with several widely held expectations: that sexual activity is inevitable unless prevented, sex is a female resource to be exploited, restrictions on sexual activity are relaxed at festivals, and masculine esteem is boosted through sexual experience. Most young people cope with these contradictions by concealing their sexual relationships [46]: as others have noted elsewhere [24,37], it is generally more important to observe discretion than restrictive sexual mores. This discretion is a pre-requisite to managing different sexual identities in different social contexts, usefully theorised by Helle-Valle [24] as 'contextualised dividuality'.

Women are greatly concerned to maintain their sexual respectability, and this norm is particularly important in relation to negotiating transactional sex. There are several widely used terms for women who are thought to be sexually disreputable, such as '***wasimbe***' (women living independently of a man or unmarried/separated mothers; singular = ***msimbe***), '*wahuni' *(which covers a wide range of socially sanctioned behaviours but in this context means 'loose' or 'promiscuous'), and, most derogatory, '*malaya*' (prostitute(s)) which refers to women who explicitly solicit sex, and who have sex with many partners with relatively little selectivity or discretion. As discussed by Helle-Valle [42](pg 387), it is helpful to recognise that the universal meaning of 'prostitute' as 'the personification of the sexually absolute [ly] immoral', may not fully apply in the same way here. In rural Mwanza, as in Botswana, the linking of sex with money or gifts is in most cases *not *regarded as immoral, and most of the transactional sex reported in this paper was not regarded by villagers as *'umalaya*' (see findings below), but rather as a normal aspect of any sexual relationship formation, continuation and sustenance.

Transactional sex as described here, differs from *umalaya *(prostitution) because of the perceived or actual selectivity of partners and the perceived moral aspect (social respectability). Like *malaya*, some other women chose to have sex with many overlapping partners over time, but they were more discreet and considered themselves selective in who they chose (e.g. not having sex with every man who asked). *Malaya *may instead primarily (or only) consider the money involved, taking it relatively indiscriminately, including having sex with men they may actively dislike. Related to this (and their perceived "immorality"), it may well be that *malaya *explicitly have many more partners and/or a higher frequency of partner change than other women.

While these factors structure the broader context for sexual encounters, they also operate at a micro-social level, shaping people's motivations to engage in sexual relationships and the negotiation that occurs within them, sometimes understood in terms of 'interactional competence' [32,47] or in terms of power differentials [10,48]. Potential sexual partners' negotiating power within specific encounters is largely shaped by their individual attributes and their immediate circumstances. For instance, a woman's physical attractiveness and a man's marital eligibility give each greater bargaining power. The extent to which women see themselves as able to negotiate sexual relationships successfully in their own interests is also likely to be critical [19].

Circumstantial factors are also important: material need, the threat of physical force or strong affection reduces a woman's bargaining power, while strong affection or intense sexual desire reduces a man's (cf. [48]). Furthermore, both parties can be disempowered by their need to present themselves differently in different social realms [24], for instance young women being potentially sexually available to seducers but having to ensure that they can conceal any sexual experience from their parents.

## Methods

The research reported here complemented a randomised trial of the *MEMA kwa Vijana *adolescent sexual health programme [49]. The *MEMA Kwa Vijana *trial showed marked improvements in knowledge, attitudes and *reported *sexual behaviour, but not in biological outcomes [49]. However, the qualitative fieldwork of which this analysis is part, did not reveal any consistent differences between intervention and control villages in the expression of sexual attitudes or reported behaviour. Any intervention-related reporting biases seem to have been overcome by the establishment of rapport over a long period through participant observation.

Data come from participant observation (PO) in nine villages conducted between 1999 and 2002, in visits usually lasting seven weeks at a time. The nine villages were selected from three districts in Mwanza region based on their trial status (intervention or comparison) and to broadly represent the range of geographic locations (roadside, interior), ethnicity and economic activities (farming, mining, and fishing) in rural Mwanza. For example, selection of villages 1 and 2 included a pair of multi-ethnic, roadside farming villages near a mine, while for villages 3 and 4 focused on remote, dispersed and almost entirely Sukuma villages. Villages 1-4 were visited for approximately two months during the same seasons each year for three years. In addition, four multi-ethnic, lakeshore fishing villages (nos 5-8) were visited only once, primarily due to limited time, and one isolated Sukuma farming village (no. 9) was visited for nine person-weeks of pilot PO only. Farming was the main livelihood in all these villages. The Sukuma ethnic group comprises 90% of the population in Mwanza region while other ethnic groups (primarily Zinza, Sumbwa and Kerewe) constitute small proportions [50]. Most villages had only one primary school and no secondary school.

Five Tanzanians and one Kenyan, aged 21 to 30, conducted the fieldwork, initially staying in villages on their own and then in mixed sex pairs of one Swahili-speaking graduate researcher (JW or GM) and one Sukuma-speaking non-graduate. The latter were selected for their previously demonstrated skills as research assistants and their fluency in Sukuma, and they were given rigorous qualitative research training. They were not residents of, and had not grown up in, the villages they were researching. Their proficiency in Sukuma meant that they could follow unsolicited conversations going on around them without having to question things, thus enhancing the validity of their findings.

The fieldwork team was trained through: informal methods seminars; detailed discussion of the main objectives of the research; reading and discussing some key methodological and ethnographic texts; accompanying senior researchers on pilot PO trips; doing a week's pilot PO; having detailed debriefings half way through and at the end of each fieldwork trip; receiving detailed comments on their fieldnotes and summary fieldwork reports; and in collectively developing a coding frame. They had an aide-memoire for broad aspects of village life such as the main economic activities, social divisions and kinship relations, as well as particularly salient aspects of social life and youth sexual culture. In debriefings they were questioned about each of these themes, but the inductive nature of ethnographic research was also stressed.

The researchers introduced themselves as conducting research on factors influencing the general health of young people, with no specific reference to sexual health. They lived in villagers' households and engaged with young people in their daily activities, in particular doing farm work and, for the women, collecting water and firewood and cooking. Young people in the host household and in contrasting households were befriended and accompanied to social events, such as markets, funerals, video shows and *ngoma *dancing, and were informally interviewed. Most PO informants were aged 14 to 25. Most had attended primary school but by the time of the fieldwork had already left, and most were not married. Data were also recorded from older adults. The researchers were encouraged to establish contacts with as representative a spread of young people in each village as possible, through the selection of their host families and by intentionally engaging with different groups and networks, e.g. religious and ethnic groups. The researchers had greatest contact with their own sex, perhaps having less licence to ignore the conventions of sexual segregation than would non-African researchers, hence the rationale of staying in villages in mixed-sex pairs. Furthermore, it was not culturally appropriate to discuss sexual issues between the sexes. The non-Sukuma-speaking researchers' key informants were good Swahili speakers. The fieldworkers wrote daily notes for one to two hours, and at the end of each field visit they wrote a summary report.

In contrast to the informal ethnographic interviews, the fieldworkers also conducted individual in-depth interviews (II) from 1999 to 2000 with 74 primary school pupils aged from 14 to 19. They were sub-sampled from a survey of pupils in 121 rural primary schools. Fifty one were chosen at random, and 23 because they had initially tested positive to HIV (20) or pregnancy (3), but subsequent tests found only 8 were truly HIV+ [30]. Thirty two were male, 42 female. The IIs were conducted by same-sex interviewers in private locations away from their home or school, usually under trees. The interviewee chose to be interviewed in Swahili or Sukuma.

The IIs took approximately two hours and were semi-structured. However, despite researchers devoting one or two hours to establishing a good rapport prior to the II, for instance by asking the interviewee to show them around the village and having a soda with them, interviewees often remained reticent and numerous prompts were required. Their reluctance to talk is probably attributable to: unfamiliarity with narrative accounts; the status difference between themselves and the interviewers; lingering mistrust about confidentiality, particularly since school pupils routinely concealed sexual relationships from adults [46]; and for young women, the importance of discretion given double standards on sexual morality [37](pg 74). The PO data are far richer than those from the IIs, and consequently this paper draws primarily upon the former.

In order to investigate particularly sensitive sexual issues such as girls' motivations to have sex, a series of discussions with same sex groups were organised in three contrasting, non-PO villages. The research teams (including GM and JW) spent three days getting to know and recruiting pre-existing friendship groups. Three or four successive discussions with the same group were then conducted by two same-sex researchers, over two to three days. Nearly all group members were between 18-21 years old, had left school and were unmarried. The discussions were tape recorded and written-up [51]. Since most of the productive and social activities were highly segregated by gender, there were very few opportunities for young people to interact with the opposite sex [46]. Hence the researchers focused on forming friendships with young people of their own sex.

The data were transcribed, translated to English, entered into QSR NUDIST software and coded by five social science researchers (including JW and GM). A coding frame of 32 broad categories had been developed by the team through each pilot testing successive coding schema with samples of PO notes and II and discussion transcripts. Each coder coded at least 1-2 documents with another coder and had their codes checked for consistency; differences were discussed until consensus was reached on how to apply the codes. JW and MP are familiar with the whole data set, having read most of the original fieldnotes and transcripts. Text coded as 'sexual relationships', 'sexual negotiation and decision making' and 'young people's lifestyles' was analysed for this paper. The data were summarised and eight main themes identified. Participants' narratives were used to illustrate these themes.

PO fieldnotes and II transcripts are identified after each quote, indicating: method (PO, II); year ('99, '00, '01, '02); intervention or comparison status; for PO only, village (nos 1-9); for II only, interview (nos 40-114); and researcher number and gender (nos 1-6, m or f). Examples are PO-00-I-3-3 m and II-00-I-76-4f. Translated extracts from fieldworkers' daily field notes are in single inverted commas in the main text, while quotes in double inverted commas are translations of informants' speech, as recorded by fieldworkers. Swahili is in *italic*, Sukuma in bold ***italic***. 'Informants' refers to those who provided information during participant observation, and 'respondents' to those interviewed.

These qualitative methods were complemented by various surveys. This paper draws on data from one, an innovative assisted self-completion questionnaire (ASCQ) administered across both arms of the trial in 1998 (N = 6079, mean age 15.1 years) [30]. Designed for a semi-literate population, the ASCQ was administered to groups of 20 primary school pupils by a research assistant who read each question in Swahili and Sukuma and then encouraged pupils to write their responses independently. The validity of these responses has been carefully examined [30]: most results were plausible, particularly with socio-demographic variables, and this method seemed more effective in collecting certain sensitive sexual information than a face-to-face questionnaire. However, there were problems with the validity and reliability of sexual behaviour data, particularly for female respondents. In this paper we therefore only refer to these survey data sparingly, to provide a crude indication of patterns across the population.

Ethical approval for this study was granted by the London School of Hygiene and Tropical Medicine Ethics Committee and the Tanzanian Medical Research Coordinating Committee. School committee chairs and head teachers provided written consent for their schools to participate in the trial, while parents were given the opportunity to refuse to allow their children to participate, and pupils were asked for written consent after an information sheet had been read aloud to them prior to the first survey. The purpose and methods of discussions and II were explained to potential participants, who provided verbal consent prior to participating. Prior to PO in each village, the researchers sought access through village leaders and then explained their research at a village meeting.

## Findings

### Background: economic circumstances, sexual relationships and survey data

Some basic information about material living conditions and sexual relationships should help contextualise the main findings. Most village houses had earthen walls and thatched roofs, and young people generally slept with same sex siblings on a crude mattress on the earth floor. They had two to three sets of clothes: for work, public places and school. Many could only wash them once a fortnight, due to lack of soap or water. Old cloth was used for sanitary towels. Most wore plastic sandals although some primary schools insisted on shoes. Few households owned a bicycle, though many could borrow one, and only around one in ten young men owned a radio.

Most girls and women walked from one to four kilometres for their household's water, depending on the season, and at least two kilometres for firewood. Typically two meals were eaten each day, usually consisting of *ugali *(maize porridge) and vegetables, and sometimes either beans, fish or (rarely) meat. However, in the cultivation season many families only had an evening meal.

Findings from the ASCQ survey across the whole of Mwanza region suggest that young men had more economic opportunities than young women. More young men reported that they sometimes/often earned money from work (62% v. 38%), that they owned livestock (31% v. 22%) and that they had their own farm plot (65% v. 59%) [30]. Both sexes could sell their labour to cultivate, both earning about 500 Tanzanian shillings (Tsh) (USD 0.83) for a full day (from about 7.00 a.m. until 6.00 p.m.), but this was seasonal work, and girls and young women usually had less time to pursue it as they had far more domestic responsibilities. Non-farming economic activities were largely patterned by the traditional gendered division of labour (cf. [43]). Charcoal burning, making bricks, transporting water and, for a few, running bicycle taxis or house building, were restricted to young men, while some young women had small businesses selling peanuts or cooking and selling food (e.g. *maandazi *(doughnuts), *vitumbua *(rice cakes), *chapatti, uji *(millet porridge) and *tangawizi *(ginger drink)). A few, generally older women (above 20), could earn as much as Tsh 1000 (1.67 USD) to Tsh 2000 (3.33 USD) a day running a food kiosk or more brewing local beer (*pombe*). Most of these activities dwindled in the cultivation season. On marriage young women generally gave up external economic activities for domestic work and subsistence farming. There were many more non-farming economic opportunities in lake-side or road-side villages than in remote areas (cf. [43]).

There was little evidence of romantic love (in Giddens sense [52]) in pre-marital sexual relationships: young people very rarely expressed the wish to get to know someone of the opposite sex, other than to negotiate having sex, and sexual intercourse did not symbolise emotional intimacy. Pre-marital sexual relationships were often short lived and pragmatic, many involving only one act of sex, and few lasted more than a few months.

However, there was evidence of possessiveness and jealousy in relationships and infidelity was one of the main reasons why they ended. There were also exceptions to the general pattern outlined above. One young man disclosed how he had had a long relationship with a young woman, involving only talking, playing and caressing, until his male friends goaded him into having sex with her. In another village young women were observed testing their love for their sexual partners through the flutter of their heart beats when they looked at them. Furthermore, some sexual relationships led to marriage.

While there was sexual mixing between villages or villages and towns (see below), there was little mixing between ethnic groups or generations. Older male partners were generally not more than 10 years older, unless in abusive relationships such as school teachers. Amongst the Sukuma, same clan relationships were taboo and very rare.

Young people expected to get married before or in their early twenties. The main criteria for choosing marriage partners were: personal and physical attraction (particularly for wives); productive potential, in terms of readiness to work hard, health and strength; respectability (particularly sexual respectability for wives); and family wealth or personal achievement (particularly for husbands). Compatibility of personalities was valued but not considered much before marriage; in general the woman was expected to accommodate to her spouse's personality.

The survey data on sexual behaviour must be treated very cautiously. Nevertheless, 52% of 14 year old boys reported that they had had sexual intercourse and 16% of girls did so. For males, but not females, reported experience of sexual intercourse was associated with earning money, even after adjustment for age. Of those who reported having had sex, 75% of females reported receiving a gift or money at first intercourse while only 43% of males reported giving something [30]. The figures relating to last sexual intercourse were 70% and 42% respectively. This may reflect a tendency for girls to have had slightly older sexual partners who could afford gifts, while some school boys were perhaps unable to do so. Only approximately one-quarter of both sexes believed that a girl was not obliged to have sex if she had received a gift from a boy. Of those who reported having had sex, 80% of boys and 71% of girls reported that their first sexual partner was either another pupil in their school or a 'youth my age but not in my school'.

### Motivations for seeking material exchange

#### Central importance of material exchange

According to most informants of both sexes, material gain was women's main motive to engage in sex. Most men thought that women had become too focussed on money in relationships, and to tell a woman '*nakupenda' *(I love/like you) had little impact unless one demonstrated this love by offering gifts or money. The majority of young women were prepared to stay in sexual relationships so long as they continued to benefit materially. It was however difficult to establish the role of sexual desire for women from the interviews since most were inhibited in discussing it, but the group discussions specifically on this topic found that young women's main motives for sexual relationships were to obtain money or gifts either out of necessity or desire [51]. Participants identified other motivations such as peer pressure, sexual desire and wishing to conceive or convince a man to marry, but these were considered relatively minor and infrequent motives compared with material exchange. Young men's main motives were to satisfy their physical desires and to gain masculine esteem, for which they were ready to exchange goods or money.

#### Poverty

In some cases transactional sex was clearly motivated by extreme poverty, to procure food, essential clothing, hygiene requirements or school necessities. During the planting season most families did not usually have breakfast; children went to school at 7 a.m. and were not expected to eat until 3 or 5 p.m. Most girls over about 14 considered that they needed a sexual partner who could give them money for peanuts or sugarcane to calm their hunger.

Parents rarely provide underwear, soap or body lotion for their children, apart from occasionally after harvest.

"My parents don't buy underwear and body oil for me, and I have to take care of this on my own". (16 yr old Sukuma woman, at school Std 6: PO-00-C-3-2f)

Consequently earning money to buy underwear was a common motive for sex:

Interviewer: How did you use that money? [received for sex]

Respondent (17 yr old Sukuma woman, at school Std 5): I used it to buy things like underpants...underskirts....

Interviewer: Mm

Respondent: And clothes to wear at home. (II-99-I-68-2f)

Many schoolgirls reported that they spent the money they received for sex on school requirements, such as books, pens, shoes, uniforms and food at school. This respondent, who was dressed in a tattered blouse during the interview, reported:

Respondent (17 yr old Sukuma woman, at school Std 5) [My boyfriend] gives me money. I buy body lotion, exercise books and pencils... A teacher may perhaps find you without shoes.

Interviewer: Mm.

Respondent: You are beaten.

Interviewer: Mm.

Respondent: Perhaps your [uniform's] blouse is torn so you have put on a dress [not uniform]: you are beaten, just like that. Perhaps you don't have exercise books: you are also beaten. Therefore I decided to do it [have sex].

Interviewer: He gave you money?

Respondent: Mm. He gives it to me. (II-99-I-68-2f)

Most young people, particularly those at school, concealed their sexual relationships from their parents for fear of serious punishment. They therefore had to disguise gifts from sexual partners, for instance by suggesting they had another source: 'W1 [a 23 yr old Sukuma woman] said she was recently bought a brand new manufactured dress for Christmas, but was not asked where it came from by her parents because she has a small business selling local beer. When they see her with new things... they assume she bought them with the profit from her beer business.' (PO-99-I-1-2f)

However, some parents tolerated their daughters' discreet relationships, and a few actually encouraged them if this helped support the household. One female informant reported of a peer:

"Sometimes when they don't have money, her mother even allows her to bring men home to make love with them to get money for expenses". (Young Sukuma woman, out of school: PO-00-I-4-4f)

There were also a few reports of grandmothers directly or indirectly encouraging their grandchildren to have sex. A young man reported of a young woman from the same village:

"If her grandmother is given a bar of soap, she allows her to go out with that man/boy". (PO-00-I-4-1 m) and two married women in their thirties described how an old woman responded when the grandchildren she supported requested soap:

"When will you ever grow up and start having men to give you money and soap?" (PO-99-I-1-2f)

Most husbands provided for their wives in the early stages of their marriage, but it was observed and reported that later most wives had to buy their own clothes, shoes and body lotion. This was despite wives rarely having the time or opportunity to earn money because of farming, household and childcare duties. Although our research focused on unmarried youth, we learnt of a few married women who had extra-marital sexual partners who discreetly provided them with material requirements. Three married women reported that they helped their friends hide gifts from extra-marital affairs so that the husbands would not find out and beat them.

#### Desire (tamaa) and peer expectations to consume

Although many young women had sexual relationships to meet immediate subsistence needs, often sex was exchanged in order to gain beauty products or clothes not essential for survival. However, the distinction between essential and non-essential items, based on supposed biological necessity, is not clear-cut (see below). Rather, it seems more appropriate to envisage a continuum from subsistence needs to consumer demands, with a large area of overlap [18].

The majority of young men said they believed that girls had sex due to *tamaa *for money (cf. [39]). '*Tamaa'*, which literally means 'longing', 'greed' or 'lust', was used in this context in two ways: to mean predominantly female desire for nice things and male desire/lust for sex [25]. Both sexes said that most men had *tamaa *for women, which was why they had multiple partners.

A strong factor shaping young women's *tamaa *for commodities, and their readiness to have sex to acquire them, were shared expectations amongst peers. By 'peers' we mean other young people of the same sex, age group and social status, that is school pupils or out-of-school youth, who were not necessarily friends. Some girls learnt from observing their older sisters and/or friends that desirable things such as clothes, shoes, scented body lotion and soap could be obtained as gifts from sexual partners. Young women said that most of them aspired to dress as smartly as any others in their village, and transactional sex was one of the easiest ways to achieve this:

"Girls entirely depend on their parents, and if a girl has desire (*tamaa) *then it becomes a problem for her, because she will desire things that her parents cannot afford, or that are not useful... This will leave her with the option of looking for men, who can give her as little as Tsh 200 [for sex]." (19 yr old unmarried Sukuma woman, out of school, food kiosk owner: PO-99-C-5-2f)

Conventional consumption was particularly focused on self-presentation. Young women in rural areas had a strong desire for nicely scented body lotion and soap, which were more expensive than non-scented products. One of the most popular brands of soap was the strongly scented GIV, costing Tsh 200 per bar, four times the cheapest soap, and it came in several different colours:

"I'm usually given money and sometimes gifts, like body lotion called Bodyline, soap called GIV, or washing soap ...". (18 yr old Sukuma woman, out of school: PO-00-C-3-2f)

Young women showed off such gifts to their friends.

Many informants noted the transformation that could be seen in girls when they started to have sex, becoming cleaner and dressing smarter. Some young women described their need to look attractive and their partner's responsibility to provide soap and body lotion, though some men had to be reminded:

"These men pretend that they are not aware that you bathe or apply [body lotion]: you have to tell them". (23 yr old Sukuma woman, out of school: PO-99-C-5-2f)

Girls and women particularly valued washing thoroughly and applying body lotion before going out in public.

"At times I am bought expensive body oil that has a nice scent, and when I apply it while going to school most pupils comment on it and admire it". (17 yr old Sukuma woman, at school Std 4: PO-99-I-1-2f)

Young women experienced considerable pressure from their peers to earn as much as possible in exchange for sex. Many reported that they discussed what they received from their partners with their close female friends. For instance, one respondent listed to a friend: "He gives me body lotion...laundry soap...and bath soap...he brought me a dress a long time ago...". Those who received little or nothing were regarded as fools for being conned. Young women judged what their peers had received from sexual partners by the type of clothes they wore and, for those still in school, by their ability to afford food. Someone who left school due to pregnancy explained:

"Before, or after, any girl has sex, she is given money by her boyfriend. If the girl is not given money, other girls laugh at her at school. Girls knew those who were not given money by their boyfriends, by seeing that they never had money and were unable to afford rice cake and other food during break time." (PO-01-I-1-2f)

School-going informants and respondents of both sexes reported peer pressure in school for transactional sex.

Respondent (16 yr old school boy, Std 7): At ten o'clock, during break, they [girls] go to buy peanuts or buns and eat. The girl [without money] will begin thinking, 'How?' By then the other girls will already know how, because in the village or centre they may have boyfriends, and they will tell the other girl: 'How long are you going to remain like this?.... We get money, we eat buns or peanuts, but you just sit there.' And therefore they advise her to do so too [have a sexual partner]. And definitely a boy will then approach her.

Interviewer: Mmh?

Respondent: Because the girl wants to be like the other girls, she therefore has to do so [have sex]. (II-99-I-42-1 m)

There was no evidence that ridiculing those who received very little from their sexual partners was a collective attempt to maintain a higher price for sex.

Some girls started off by borrowing clothes and lotion from their friends and then, once they had attracted sexual partners, they were given their own and could maintain their supplies. However, peers were rarely prepared to continue sharing their possessions for long with someone who did not start earning them herself by attracting a sexual partner.

Due to their *tamaa*, girls and women were said to agree readily to have sex with those perceived to have money. A 23 year old woman was heard telling another woman 'that she would rather have sex with a man who has two wives, but who could give her a piece of soap when she needs it, rather than having sex with young men who do not have wives but would give her nothing.' (PO-99-C-5-2f)

The motivation to be given money or gifts for subsistence, and to get them for non-essential consumption, were very difficult to distinguish. Neither women nor men made this distinction themselves. Rather, women tended to present their motivation in terms of subsistence needs (cf. [18], and men attributed women's motivation to consumer desire (*tamaa*), the two perspectives clearly reinforcing respective bargaining positions. In practice the two motivations were sometimes inseparable since for a young woman to attract a sexual partner to meet her subsistence needs, she might have needed the clothing and beauty products to look attractive.

This was illustrated by the importance of scented soaps and lotions, which had several inter-related uses: to avoid diseases associated with poor hygiene, clean oneself, look smart, impress female peers, remain attractive to one's sexual partner, attract new sexual partners or wash after having had sex.

"Now an ***msimbe ***like me, a man cannot cheat me. Does it mean I don't wash or apply lotion? He must give me money for soap and body lotion, so that when I come from there [having sex with him] I can wash." (19 yr old Sukuma woman, out of school: PO-99-C-5-2f)

#### Gaining capital for small businesses

A few enterprising young women engaged in transactional sex to accumulate capital to start a business, such as trading food products, preparing and selling snacks, or operating food kiosks. They considered this a wiser use of money received from sex than buying commodities. A 19 year old ***msimbe ***described how: '...When a man gave her Tsh 2,000, she added it to what she had got from others [sexual partners] and started her food kiosk business. She said a woman has to be clever in spending the money given to her by men. Otherwise you will always spend everything they give you and end up borrowing every day.' (PO-99-C-5-2f)

However, while transactional sex sometimes enabled young women to start a business, such businesses may have also provided opportunities for transactional sex. In fact, for some young women the businesses were valued as an excuse to get home late, allowing them to meet sexual partners. They also provided an easy way to explain to strict parents how new clothes or money were acquired.

#### Farm labour

One village was untypical in being within walking distance of large, company-owned cotton plantations. A few female informants described walking long distances to work there in mixed sex labouring groups, paid by piece work. The young men frequently completed their allocated work before the women, and then offered to help them in exchange for sex and/or money. The women, often exhausted by the long hours and heavy work, and risking being paid nothing for an uncompleted row, usually accepted. At the end of the day they either paid their assistant or had sex with him in the bush.

"Males and females cultivate together on a large cotton plantation at B. Usually boys complete their portion before girls and some offer to help them. Some women agree ... and immediately after cultivation, they enter the bushes and have sex there before going home." (19 yr old woman, out of school: PO-01-I-1-2f)

Those women who returned to work on the plantations realised that they were likely to need such male assistance.

#### Symbolic dimensions and eligibility for marriage

Material exchange for sex sometimes had symbolic as well as direct economic significance. Men sometimes demonstrated their affection for a sexual partner through the generosity and consistency of the gifts and money they offered, and a man who loved a woman was expected to provide gifts or money without prompting. Material exchange was also assumed to buy exclusivity, unless the woman/girl was renowned as having multiple partners, in which case a lower price was probably paid (see below). If the man/boy discovered his partner had been having sex with others he was likely to end the relationship.

Transactional sex was as important in relationships that (at least) one party hoped would lead to marriage, as it was in short term relationships. In fact, the more gifts or money men provided the more desirable they were as potential marriage partners. The size and frequency of gifts could be deemed to reflect a man's long-term ability to support a woman, and the fieldworkers learnt of marriages entered into on this basis. However, they observed that, once married, many men shared less money with their partners.

### Negotiation

The gifts reported to be commonly given in sexual encounters were sugarcane, peanuts, soap, body lotion, underwear and underskirts. If money was exchanged it was generally between Tsh 200 to Tsh 1,500, though occasionally the range extended from Tsh 100 to Tsh 5,000. Several informants reported that the amount acceptable had recently fallen due to general economic circumstances:

"For many, now that money has become scarce, even Tsh 200 is enough [laugh]." (Young woman: PO-99-C-8-2f)

The type of gift or amount of money exchanged varied from one encounter to another according to negotiation. We will first describe the process of negotiation and then the factors affecting the actors' bargaining power.

#### Process of negotiation

Explicit sexual negotiation was almost always initiated by men, although females may have actively encouraged it. The statement '*nakupenda' *('I love/like you') was often accompanied with some reference to material exchange, since men believed this to be a good way to persuade a girl/woman to have sex:

Interviewer: What are the first words you use when making arrangements for making love?

Respondent (16 yr old school boy, Std 7): The main words which are used to induce a girl is simply to give her money.

Interviewer: Mmh?

Respondent: I tell her: 'If you agree, I shall give you a certain amount of money'. And this is due to her lust for money, in order to buy some necessities so that she can look like her friends. (II-99-I-42-1 m)

According to many women, men sometimes asked a girl/woman to specify how much money she wanted for sex immediately after they approached her.

If the man did not mention gifts in his seduction some girls/women raised it themselves. For example, women frequently asked for a soda, not meaning literally a soft drink (which would cost Tsh 200), but indirectly asking for money without feeling embarrassed or acting like *wahuni*.

"Women have become money-minded so when a man tells them, '*Nakupenda*', the woman responds, 'Then leave me with something for a soda'." (Young Sukuma man, married: PO-01-I-1-2f)

Most women said that the onus was on them to remind their sexual partners of what they wanted before they agreed to sex, as illustrated by this same male informant who said that when he visited his girlfriend at night:

"Immediately after I entered she asked me, 'Where is my soda?"' (PO-01-I-1-2f)

He told her that he had not brought any soda but instead gave her Tsh 1,000: they had sex that evening. Some women asked for money indirectly, for instance by hinting at the need for a loan. Others did not refer to material exchange at all but delayed agreeing to have sex until they were given, or were promised, money or gifts.

Sexual negotiation sometimes involved explicit bargaining between potential sexual partners.

"When I told the girl that I wanted to have sex with her, she told me to give her Tsh 2,000. I told her that I did not have that amount of money and the girl said I should then give her Tsh 1,500. I told the girl that I did not have that amount, but I could give her Tsh 700. The girl said that the money was not enough, and after long negotiations we agreed that I should give her Tsh 1,000". (Young man: PO-01-C-2-1 m)

Payment was not always made before sex took place. For example, when a girl asked a male informant for Tsh 1,000 before they had sex, he told her:

"I don't do such business, I only give money after the act [having sex]". (PO-99-C-5-2f)

In this case he did pay as requested. In on-going relationships sex was sometimes provided on credit, young women being understanding of their partners' financial difficulties.

"If the boy doesn't have money and the girl demands it, the boy asks the girl to lend it to him, that is, he makes love with her and gives her money on another day. In fact after some days or a week, the boy pays the girl her money. Usually due to adverse life situations, girls are given very little money, like Tsh 50, Tsh 100 or less than Tsh 1,000". (Young man: PO-00-I-4-4f)

It was generally considered disreputable for a woman to explicitly solicit sex. Formal prostitution *(umalaya) *was condemned and women suspected of it were the subject of gossip. A *malaya *was a woman who openly had sex with many partners in exchange for money/gifts, not maintaining the discretion and at least appearance of monogamy of respectable women.

#### Intermediaries: "Posta"

The vast majority of young men reported that they had, at some point, involved intermediaries in their sexual negotiation (also common in Kilimanjaro [25]). Intermediaries were referred to as *posta *(literally 'post office' or 'mail') and were individuals close to either the male pursuer or the woman being sought (e.g. close relatives, friends, or fellow members of work teams). The *posta *encouraged the girl/woman to like a particular man, and facilitated him giving her gifts. The female researchers were themselves sometimes approached by *posta*, as happened to this 25 year old fieldworker: '... she asked me whether I would also like to have a lover. She told me that if I took a lover, he would help me meet my daily requirements.' (PO-99-C-5-2f)

*Posta *could weaken or strengthen the girl/woman's bargaining position, which usually depended on his or her relationship to the two parties. If related to the boy/man, the *posta *was likely to encourage the girl to agree to sex for only limited money or gifts, while if related to the girl, the *posta *was likely to help her negotiate more money or gifts.

*Posta's *efforts, and others who helped facilitate an encounter, were sometimes rewarded with small gifts from the man. The fieldworkers noted several examples of a younger sibling/relative being given money for keeping silent, unlocking a door for a girl to return from having sex, and/or having to share a room in which a couple had sex.

#### Relative bargaining power: attributes

The amount of money or kind of gift exchanged was shaped by the relative bargaining power of the sexual partners. Several factors influenced this, relating to each partner's general attributes and particular circumstances.

The main attributes that made a girl or young woman desirable, and therefore able to demand greater remuneration, were her physical attractiveness, in particular light skin colour and large buttocks, her appearance in terms of clean, smart clothes and elaborately made up hair, her respectability and her prestige as a newcomer.

The greater a young woman's respectability, the more desirable she was to her potential partner and the more she had to lose by agreeing to sex, which usually meant that more valuable gifts were exchanged. Meanwhile one of the characteristics of less sexually reputable women, primarily ***wasimbe***, was that they were said to be less discriminating about their sexual partners, caring less about their age or marital status, and they agreed to sex for very low prices (as little as Tsh 100). Women defined by men as "bad" for being disrespectful (*kiburi*) or *wahuni*, attracted less sexual partners and received very little for sex. Furthermore, young women who agreed to sex for minimal reward were sometimes suspected of having AIDS. 'He said that it seems that the girl was aware that she had AIDS since ...when she came to [Village 4] she was ready to have sex for small amounts of money between 200/- to 300/- shillings.' (PO-02-I-4-1 m)

Nearly all informants reported that newcomers to the village, of both sexes, were perceived as highly attractive sexual partners. These newcomers may have been complete strangers or may have emigrated from the village but occasionally returned on celebration days. Having sex with a newcomer was prestigious, especially if s/he had come from a town. It was observed and reported that urban girls and women usually had 'good clothes', had 'nicely made hair' (chemically softened), and were cleaner than most village women. Men competed with each other to be the first to have sex with a female newcomer, sometimes offering her as much as Tsh 5,000. The four female researchers experienced this themselves during participant observation, with some men offering as much as Tsh 10,000 for sex. However, the sexual attractiveness of newcomers was likely to wane as soon as they were known to have been seduced locally, and if they came to be seen as having had several partners they rapidly acquired the status of ***wasimbe***, with limited bargaining power.

The most important attribute shaping the desirability of a male partner was his perceived earning capacity. Schoolboys had the least to offer, in contrast to young men with small businesses or who were farming, and adult men.

"Most girls prefer villagers [not at school] to school boys [as sexual partners], because villagers give them more money than school boys. Schoolboys give Tsh 200 and at most Tsh 500, while a villager gives Tsh 1,000 to a girl." (17 yr old Sukuma woman, at school Std 4: PO-99-I-1-2f)

Furthermore, a few girls said that schoolboys not only offered very little for sex, but frequently failed to pay what they promised. Men with little income were likely to have few partners.

It was reported that the men most favoured as sexual partners were those with a steady, relatively high income, such as teachers, government employees (e.g. village authorities) and business people, and a few women reported relationships with such men. Young women with sexual partners who owned shops or kiosks said that they went there to select whatever item they wanted without paying, or to ask for money.

Personal attractiveness, whether in terms of "good behaviour" (*tabia nzuri*) or appearance, seemed to be less important for a man to win sexual partners than "the weight of his pockets". There was a common saying that: "There are no unattractive/ugly men, and if there are, then they are those without money". However, if a man was perceived to be attractive a young woman might accept a more modest payment for sex, while if unattractive he was likely to be refused unless he offered a lot more. The following fieldnote shows how girls sometimes demanded a price clearly intended to avoid sex, though we cannot tell why: 'K2-m (a 23 yr old man) said they were coming from the well where they had followed some girls. ...the girls had told them that if they wanted to have sex with them, they should give them Tsh 10,000. They said they were not ready to give the girls that amount of money to have sex with them only once...they could have sex with lots of other girls for a small amount of money.' (PO-00-I-4-1 m)

Like female newcomers, male newcomers to the village were particularly valued as sexual partners. This was primarily because of their perceived affluence, but also the prestige of their presumed modern urban lifestyle. The male researchers were thus also sought as potential sexual partners, although less directly than were the female researchers, for instance women tried to get the female researchers to act as intermediaries to access male researchers. Women known as ***wasimbe***, or reputed to be *wahuni*, preferred newcomers who were ignorant of their sexual reputations which might have undermined their bargaining power.

There were occasional evening video shows in the villages, brought from the district capitals by young men. Some young women got a man to pay their Tsh 100 entrance fee, agreeing to have sex afterwards with their benefactor. Frequently this was one of those running the show:

"The young men who come to show videos in the village usually have sex with village girls, because they allow them to enter free of charge and are also seen as having money and thus being potential providers." (Male attendee at video show: PO-99-I-1-2f)

#### Relative bargaining power: circumstances

Whatever their attributes, partners' current circumstances also shaped their bargaining power, in particular women's immediate material needs, men's access to ready cash, and the stage of their relationship.

In general, the more affluent a girl or woman was, the greater payment she was likely to demand before agreeing to have sex. As an ***msimbe ***observed, to the affirmation of another:

"If a man realises that a girl has no source of income he takes advantage of her and can even have sex with her for Tsh 100.... Men take advantage of a woman who does not have her own source of income. If you just wait for men they make fun of you, like goodness knows what. They will really show you contempt. It is better that you have your own small source of income, and if a *hawara *(lover) adds to your capital, you just continue with it". (19 yr old Sukuma woman: PO-99-C-5-2f)

However, the amount given for sex was also shaped by the male partner's access to money and the girl/woman's bargaining skills. Schoolgirls in their early teens usually only received sugarcane or peanuts from sex with schoolboys, as a male recent school leaver said: "I mean, there at school, girls are finished for just peanuts." However, older girls, and girls who had out-of-school lovers, could demand and receive larger gifts, partly because their partners had more money, but in some cases because they argued that they had less parental support and therefore greater needs.

The dry, harvest period was a festive time with traditional drumming, dancing competitions and little farm work. Farmers were at their most affluent shortly after harvest when they sold their cash crops, and their available money then dwindled until the next harvest, unless they found employment as labourers during the planting season. Consequently the frequency of sex, and payment accepted for sex, varied seasonally. During the rainy season, it could often be as little as Tsh 200, while after harvest this frequently increased to as much as Tsh 1,000.

Similarly, fishermen were more likely to agree to pay large sums for sex when they had just returned from a fishing trip and had ready cash. In lakeshore villages a few young women targeted fishermen when they had just returned from a catch, by strolling about on the beach in tight fitting dresses. These women were said to '*kuchuna mbuzi'*, literally 'skin the goat', meaning lure the fishermen into having sex for money.

As a relationship developed, bargaining power tended to increase for the man/boy. It was widely reported that more money or gifts were necessary to seduce a girl or woman into starting a sexual relationship than to have sex on subsequent occasions:

"In the beginning of a relationship the man gives relatively more money. But as the relationship continues he reduces it, or even becomes sly so as to have sex for free... [When] I met my boyfriend for the first... [sexual encounter] he gave me Tsh 1,500; this was reduced to Tsh 1,000 the second time." (Sukuma schoolgirl, Std 7: PO-01-I-1-2f)

Several reasons may combine to explain why less was exchanged for sex after first intercourse within a relationship (also found in Durban, South Africa [8]). The young woman might have become less desirable to her partner, the prestige of seduction having been achieved and fantasies of sexual contact realised. Meanwhile the woman's respectability had been undermined by her succumbing to his pressure to have sex. Furthermore, most men were unable to maintain the gifts offered for first sex. Several young people reported that consequently young women often ended relationships after a few sexual encounters, to find more lucrative ones:

Respondent (14 yr old Sukuma school girl) It was me who rejected him.... I was asking for money and he refused, I asked for body oil and he refused

Interviewer: Now, that [new boyfriend], does he give you money?

Respondent: He gives me money. I buy body oil, exercise books and pencils

Interviewer: About how much money does he give you?

Respondent: Tsh 1,500. (II-99-C-51-2f)

Exchanging sex for gifts or money thus gave women an incentive to change partners, although it encouraged men to keep them.

#### Bullying, tricks and deception

There were various ways in which men sometimes manipulated women or girls' circumstances to strengthen their bargaining position. Physical coercion in sex was generally condemned, and sometimes resulted in fines by the village authorities, but cases rarely reached that level, and then usually only if there was evidence of extreme violence, the girl was very young, and/or there was no prior gift-giving [53]. It was difficult to ascertain the prevalence of coercive sex, but threats seemed more common than actual violence, which was primarily associated with heavy drinkers. Threats were generally made when a young woman was thought to be reneging on an explicit or implicit agreement to have sex in return for a gift. For instance, young women reported that if they refused to have sex after consuming a gift at festivals, men often threatened to beat them or snatch away their *kanga *(cloth tied like a skirt). They said that they agreed to have sex to avoid such embarrassment.

Men were said sometimes to bully women into agreeing to sex by fabricating stories about prior gifts. One female informant reported:

"He gives her, like, Tsh 20 for sugar cane, then he wants to beat her if she refuses [sex]. If asked by people why he wants to beat her, he says she ate [took] his money worth Tsh 2,000". (13 yr old Sukuma schoolgirl, Std 5: PO-99-C-5-2f)

Young women sometimes agreed to sex in such cases to avoid embarrassment and protracted disputes.

A common trick was for a man to use an intermediary to get a young woman to accept a gift indirectly. For instance, a few food kiosk owners reported that men sometimes told them to serve food to a particular woman, suggesting it was the kiosk owner's gift. The man then paid the bill, putting him in a strong position to negotiate having sex with the woman, since she was indebted to him. However, most women were aware of such tricks:

"A man buys you tea or soda, he gives you money, he cunningly pleases you. He can cater for your costs first before propositioning you." (Young Sukuma woman, out of school: PO-99-C-8-2f)

Some girls and women described being deceived with false promises of future payment, though if this was repeated several times they would seek a new partner. A young female informant bitterly described her boyfriend as "sly":

"He tells you, 'Tomorrow, when tomorrow comes.' He says, 'Tomorrow', like that, and you continue to have sex with him. Eventually a month is over without being paid your money". (PO-99-C-5-2f)

Failure to fulfil such promises could lead to considerable animosity, especially in situations where the young woman's relatives knew about it, and particularly if they benefited from the sexual exchange. Men were sometimes compelled to honour promises of payment, sometimes surreptitiously:

"When a boy agrees he will give a girl money if they make love, and then afterwards he doesn't give it, that girl goes to tell her grandmother. The grandmother gets very annoyed that the boy makes a fool of her grandchild by not giving her anything. The grandmother looks for local medicines to make the boy 'become a woman' [impotent], that is ... he loses the power of making love.... After some time the man discovers the change and immediately goes to a traditional healer for divination. The traditional healer tells him the truth, that he did not give the girl her money after making love with her. That boy goes to seek forgiveness from the girl and gives her much more money than they had agreed previously." (Young woman: PO-00-I-4-4f)

### Festivals

The Christian holidays and national festival days were important in the villages and eagerly awaited, villagers saving up money and gifts for these occasions. Much greater license than normal was given to young people, and young women with strict parents had a rare opportunity to hang about in the evening and talk to friends.

Young women were very concerned that their clothes should be as smart and new as possible on these celebration days. They often used their savings to this end, or if necessary borrowed from their friends or boyfriends. For instance as Christmas approached a 17 yr old schoolgirl (Std 5) 'was thinking of asking her boyfriend to give her Tsh 1,000 so that she can add to what she has and go to Geita [district capital] to buy shoes' (PO-99-I-1-2f), or in July a young woman said:

"I was given money by my partner in order to buy socks for the choir at the *Sabasaba *inauguration". (PO-00-I-4-4f)

On these days there was more sexual negotiation and activity, and transactional sex was much more explicit, usually taking the form of "*halleluya*" exchanges, deriving from 'alleluia' meaning praise and thanks to God. If someone greeted another person with the word "*Halleluya*" they expected to be given a gift or money. Most young women preferred to ask men for *halleluya *gifts because they gave more, but the women knew that they were likely to have to reciprocate by having sex, since most quickly spent the money or consumed the food or drinks given. Men generally demanded to have their gift back, or to have sex with the recipient, once they were sure that she had used it; if she still had the gift or money she might return it and avoid sex.

Many men in the market place demonstrated their affluence by buying sodas or beer conspicuously to attract women. Men often used festivals to seduce previously unobtainable women, offering them larger *halleluyas *than those who were easier to seduce.

Market days were held once or twice a week, depending on the village, attracting people from nearby villages and towns. Most operated in the evenings, allowing pupils to attend, for whom they were important social occasions. Many young people reported that they met their sexual partners there and that much sexual negotiation and exchange happened there, sugarcane playing a prominent role.

### Transactional sex and HIV/AIDS risk

Nearly all young people had heard of AIDS and understood that it can be contracted through sex, though few had heard of HIV. Knowledge about disease progression was limited, and villagers still thought of AIDS as a primarily urban disease of the sexually immoral [54].

Material exchange for sex probably increased the risk of transmission of HIV and other STIs in four ways. Most obviously, it was young women's main motivation to have sex and therefore it encouraged sexual activity that might not have occurred at all if young women had had other sources of income or means of acquiring goods. Second, it provided a dynamic for partner change, as discussed above. Third, it meant that men's desirability as sexual partners was closely related to their perceived affluence. Given the current distribution of HIV [55], the most desirable men were more likely to be infected, since they could afford to maintain more than one partner, to change them frequently, or they come from the towns.

"Because if it is known that a boy has some source of income, the girl will definitely [have sex]... Because she will know that this boy has money and she will be sure of getting that to fulfil her needs...Therefore...she will choose you and not someone else who she knows does not have anything that will help her." (Male: II-99-I-42-Im)

A fourth link between transactional sex and HIV was that it could create a further barrier to condom use. Use of condoms was said to be uncommon in the villages, most young people reporting that they had never used them before, for several reasons, including partner refusal [56]. According to young people most men considered that they were not getting good value for their money with a condom:

"Most men who give money to their lovers don't agree to use condoms. When it happens that a woman insists on using a condom, they refuse and demand to be paid back their money". (15 yr old Sukuma schoolgirl, Std 4: PO-00-C-3-3 m)

They reported that such young men often become angry and wanted to '*komoa' *(humiliate) women who insisted on using a condom after taking money.

## Discussion

The evidence from intensive participant observation in rural Mwanza suggests that material exchange for sex was very common and that it underlay most non-marital relationships, as previously suggested from group discussions [16]. While these relationships themselves were often condemned by older adults, there was no evidence that the linking of sex with money or gifts was regarded as immoral (cf. [42]).

Traditionally rights over children, access to sex, and other important rights were exchanged for bridewealth. It may be that the illegitimacy of sex before some form of marriage, illustrated in the euphemism '*kitendo cha ndoa' *(an act of marriage) for sexual intercourse, stemmed from the lack of payment to the woman's family for access to sex. Material exchange for pre-marital sex could then be seen as a modification of conventional norms: the contentious issue was not the material exchange but that the woman's family did not benefit, or may have lost potential income if she was considered less marriageable because known to have had premarital sex. Whether norms around transactional sex relate to bridewealth could be studied through comparisons between ethnic groups with different bridewealth practices, and investigating those matrilineal societies where bridewealth is not required.

This focus on material transactions in sexual relationships is not meant to imply that other motives for sex did not exist. Indeed, we have noted that for young women a man's physical attractiveness, his social prestige or simply affection could all override material considerations. Furthermore, gifts sometimes symbolised the man's love or/and long term ability to support his partner. However, our data do not suggest that the material exchange was primarily symbolic, as argued in a study with Ugandan secondary school pupils [15]. In most of the pre-marital sexual relationships learnt of through this participant observation with primary school and out-of-school youth, the material benefits of sex were a prime motivation for young women. To have sex and *not *to seek material exchange would have had serious symbolic implications for them, suggesting they were sexually available to anyone and did not value themselves.

The relationships presented here do not quite fit any of those that Setel identified in Kilimanjaro, despite the sophistication of his typology ([25]: Table four point one). They come closest to what he terms '*starehe*' (recreational) or '*nipe-nikupe*' ('you give me, so I give you') relationships, but the woman's desire for her partner was less important than Setel describes, while receiving gifts was more significant than he suggests for '*starehe*' relationships. We found very little evidence of Setel's most romantic category, '*urafiki*' or '*mpenzi*' relationships. It is also interesting to contrast these rural Tanzanian findings with those from Durban, South Africa, where gift giving appears to play an important and accepted role in young people's sexual relationships, but monetary exchanges are condemned as a form of 'prostitution' [8]. This suggests that the sphere within which money is a legitimate form of exchange may have become more restricted in that more industrialised society [57].

**Figure 1:**
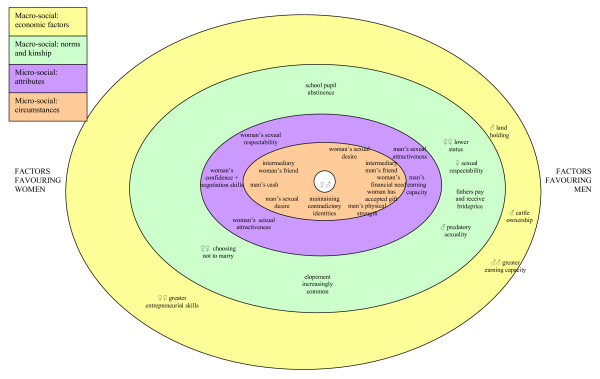
**Simplified representation of factors favouring men (to seduce women and/or minimise gifts) and women (to resist or seduce men and/or maximise gifts) in their sexual negotiation**. This combines findings from rural Mwanza and the published literature on Tanzania.

Young women's relative power in sexual relationships looks different at macro and micro-social levels, as Luke [10] found in her review of economic asymmetries in sexual relationships. Figure 1 attempts to illustrate this diagrammatically, although it inevitably simplifies the complexities of sexual negotiation. The main macro-level factors shaping transactional sex overwhelmingly benefited men. These were the distribution of economic power (outer ring, Fig. 1), even though employment changes have undermined men's power [38], and kinship and normative factors (both in second ring, Fig. 1), in particular the lower status of women and the main features of sexual culture. At a micro-level, however, there were different dimensions of power, some of which generally benefited men (e.g. physical strength), while others tended to benefit women (e.g. sexual attractiveness or their prestige as sexual partners) (cf. [48]). The factors shaping the relative bargaining power of (potential) sexual partners can be divided between their individual attributes (third ring, Fig. 1) and the specific circumstances of a particular sexual encounter (inner ring, Fig. 1). Individual economic circumstances affect both sexual partners' bargaining positions.

This study shows that young women actively used their sexuality as an economic resource, and, within the constraints of macro-social factors, they often willingly entered into relationships primarily for economic gain. Although women may have been pre-occupied with meeting their immediate needs through transactional sex, there were signs that some of those in their late teens and early 20s also looked at their relationships in terms of long term benefits. This is seen in their choice of partners who could provide both in the short and long term as marriage partners, or in case of unplanned pregnancy could take care of them and/or the baby. We therefore argue that some young women were strategic in their choice of sexual partners, being more attracted to those they thought had financial potential. In contrast, elsewhere in northern Tanzania, women's strategic calculations to maximise their long-term economic interests lead some to have serial partners rather than get married [40]. Such a long term perspective was not explicitly evident with the younger women in this ethnography, but this might illustrate an earlier stage in women's relationship careers, in which they first learn to employ their sexuality but might end up in marriage, although this may not have been their primary goal for entering the relationship.

The different factors shaping the relative bargaining power of sexual partners led to apparent contradictions concerning the amount exchanged for sex. For instance, sex with school girls was either relatively cheap, because they welcomed even very small gifts, or relatively expensive because they were perceived to be virtuous and usually unattainable to adult men. While in general greater material need constrained a girl or woman to have sex for less, some girls demanded more from their partners on the grounds that they did not have financial support from their parents. Or whereas some newcomers were perceived to be highly desirable, and could therefore command a high price, others were assumed to have had many partners and so received less money. A limitation of this study was that there were no individual case studies and insufficient data were collected on the different factors operating in specific negotiations, a challenging task without being present. However, the apparent contradictions could probably be explained through the different negotiating and presentational skills of those involved.

The material needs that prompted women to have sex ranged from hunger to wanting a new dress for a festival (as in South Africa: [3,18]). However, we consider it fruitless to attempt to distinguish between 'absolute poverty' and 'relative poverty', with the implication that transactional sex is somehow more legitimate with the former [9]. First, it is likely that people's primary experience of poverty is being excluded from their normal social life [58], so lacking a new dress at Christmas when one's peers have one was experienced as poverty, even though not life-threatening. Second, in practice, for a young woman to attract a sexual partner to meet her subsistence needs she generally required the commodities to look attractive (cf. [3]). Young women engaged in transactional sex at any point in the limited range of poverty to (relative) affluence within villages. It appears to be a Western-centric ethic that seeks excuses for the explicit linking of sex with material gain [20], although the young women nearly always explained the practice in terms of a discourse of 'needs'. Similar justification of sex for luxuries amongst Durban township women has been interpreted as a defence against accusations that they are sexually exploiting men [18].

Transactional sex was also reinforced by the norm of reciprocity [59]. This seemed to underlie women's indignation when access to sex was not paid for, or men's when a gift was not returned in sexual favours. Although the principle - that a gift is never free but involves an obligation to provide something in return - does not necessarily require immediate reciprocity, in many of these young people's sexual encounters little delay was tolerated. Indeed, where a partner accepted a long delay in reciprocation it demonstrated a more enduring relationship.

Villagers' views about transactional sex illustrated the contradictory norms around young people's sexual behaviour in Mwanza [46]. Some parents exercised discretion and kept different social realms separate [24], condemning pre-marital sexual relationships but also telling their daughters to support themselves, knowing how this was done in practice, or they readily shared the gifts provided by daughters without enquiring from where they came. Relatives' tacit collusion in this way could enhance a young woman's negotiating position. Conversely, women's negotiations were severely constrained by the need to maintain their reputations, even though transactional sex was almost ubiquitous. To explicitly ask for payment was disreputable, but so too was receiving nothing for sex, which cheapened one. Consequently most women protected their reputation by not asking for gifts or money directly, but delayed agreement to have sex until they were given or promised something. An important health consequence was that this prevented an explicit agreement that payment was for sex with a condom. Similar problems have been found in the West [60].

Material exchange for sex increased the risk of HIV transmission and other STIs more directly, for the four reasons identified above: encouraging greater sexual activity; providing a powerful dynamic for partner change as new partners tended to give more valuable gifts; making affluent men, who were more likely to be infected, the most attractive sexual partners; and creating a further disincentive to use condoms, since most men considered that they had not got "value for money" if they had to use one. Other qualitative studies have found that women are less likely to demand condom use if it jeopardises their material gain (e.g. [3,17,18,61,62]), and Luke's [10] review found that economic exchange is associated with unsafe behaviours. This is one of the main explanations that Dunkle et al[2] offer for the association they found, in a large study of South African antenatal clinic attenders, between transactional sex with non-primary partners and HIV seropositivity. However, it should be borne in mind that in rural Mwanza few young people of either sex wished to use condoms, irrespective of sexual exchange [56]. Partner violence is not intrinsically linked to material exchange, but we found it was occasionally provoked by disputes over negotiation. In South Africa partner violence has been associated with transactional sex and HIV seropositivity [2].

Preventive interventions are more likely to be effective if they acknowledge the economic importance of sex for young women [3,18]. Alternative income-generating schemes may reduce the transactional sex that is motivated by poverty. In addition, such schemes might help those girls who continue to engage in material exchange to negotiate higher prices and to resist high risk sexual partners or unprotected sex, if these risks were salient to them.

However, given how embedded transactional sex is in this culture, its role in the construction of respectable selves, and the dearth of alternative options for girls in particular to distinguish themselves, providing other economic opportunities is unlikely to end transactional sex without deep cultural change. Furthermore, individuals with less immediate need for money, as a result of alternative sources of income, may only be willing to have sex with more affluent men who may, at least at this stage of the epidemic, be more likely to be HIV-infected.

If, recognising this, harm-reduction approaches were adopted, they might encourage young women to negotiate condom use at the outset, or to be more selective of partners in order to safe-guard their reputations, allowing them to demand a higher price and thus have sex less often. At minimum, educational programmes should develop communication skills that include scripts relating to gift giving and the expectations and intentions involved in gifts [8]. Any of these approaches would need careful piloting and evaluation.

## Competing interests

The authors declare that they have no competing interests.

## Authors' contributions

JW conducted the field work, qualitative data analysis and drafted the manuscript. DW designed the study, conducted analysis and participated in writing the manuscript. MP participated in the coordination and write up. GM participated in the data collection and write up. DR participated in the design of the study and write-up. All authors read and approved the final manuscript.
